# Persistent immune, coagulation and cardiac dysregulation are correlated with later post-discharge mortality in children with severe malnutrition

**DOI:** 10.1186/s12916-026-04647-9

**Published:** 2026-01-22

**Authors:** Brenda Kamau, Evans O. Mudibo, Cecillia Wechessa, Elisha Omer, Bonface M. Gichuki, David M. Mburu, Laura Mwalekwa, Molline Timbwa, Johnstone Thitiri, Moses M. Ngari, James A. Berkley, James M. Njunge

**Affiliations:** 1 KEMRI-Wellcome Trust Research Programme, Kilifi, Kenya; 2 The Childhood Acute Illness and Nutrition Network, Nairobi, Kenya; 3 Division of Human Nutrition and Health, Wageningen University and Research, Wageningen, Netherlands; 4 Host-Microbiota Interactions Lab, Wellcome Sanger Institute, Hinxton, UK; 5 Pwani University, Kilifi, Kenya; 6 Coast General Hospital, Mombasa, Kenya; 7 Mbagathi County Hospital, Nairobi, Kenya; 8 Center for Tropical Medicine and Global Health, Nuffield Department of Medicine, University of Oxford, Oxford, UK; 9 Gonville and Caius College, University of Cambridge, Cambridge, UK

**Keywords:** Complicated severe malnutrition, Post-discharge mortality, Inflammation, Immunosuppression, Coagulation, Endothelial activation, Sepsis

## Abstract

**Background:**

Children with complicated severe malnutrition (CSM) face high mortality after hospital discharge, yet the underlying mechanisms remain poorly understood. While early post-discharge mortality (< 2 months) has been linked to a sepsis-like inflammatory profile measured at discharge, it is unclear whether this relationship persists (later mortality; 2–6 months post-discharge). This study investigated whether immune, inflammatory, and endothelial dysfunction at 2 months post-discharge are associated with later mortality in children recovering from CSM.

**Methods:**

We conducted a case–control study nested within a randomised placebo-controlled trial of daily co-trimoxazole in HIV-negative children aged 2–59 months with CSM in four Kenyan hospitals. Cases were children who died between 2 and 6 months post-discharge; controls were survivors frequency-matched by sex, site, and trial arm. Plasma cytokines, chemokines, endothelial markers, and untargeted proteomics were measured at discharge and 2 months post-discharge. Conditional Cox regression, adjusted for age, sex, site, mid-upper arm circumference (MUAC), and randomisation arm, was used to identify biomarkers associated with later mortality.

**Results:**

Cases were younger (had a median of 7 vs. 11 months), had longer hospital stays (14 vs. 10 days), and showed lower anthropometry (MUAC = 10.7 vs. 12.0 cm) and lower haemoglobin (9.7 vs. 10.6 g/dL) at 2 months post-discharge (all *p* < 0.05). Mortality 2–6 months post-discharge was associated with elevated inflammatory mediators (e.g. IL-10 [hazard ratio, HR: 1.47, 95% confidence interval, CI: 1.00–2.14], IL-15 [1.65, 95% CI: 1.08–2.51], IFN-α2 [1.51, 95% CI: 1.02–2.23]), acute phase proteins, apolipoproteins and coagulation markers, including fibrinogen, histidine-rich glycoprotein (1.40, 95% CI: 1.01–1.94), protein C inhibitor (SERPINA5, 1.50, 95% CI: 1.07–2.08), SERPINA10 (1.42, 95% CI: 1.02–1.99), and ADAMTS13 (0.41, 95% CI: 0.24–0.70). Additionally, cardiovascular and muscle-related proteins such as angiotensinogen (1.46, 95% CI: 1.03–2.08), α- and β-tropomyosin (0.68, 95% CI: 0.48–0.98), PI16 (0.72, 95% CI:0.54–0.97), and zyxin (0.61, 95% CI: 0.40–0.92) were elevated in cases.

**Conclusions:**

Later mortality in children recovering from CSM is associated with persistent immune activation, a sepsis-like phenotype involving multiple systems. These findings suggest that children at risk of later mortality may benefit from biomarker-guided interventions initiated at discharge.

**Supplementary Information:**

The online version contains supplementary material available at 10.1186/s12916-026-04647-9.

## Background

Certain groups of children in sub-Saharan Africa and South Asia have a high risk of mortality after hospital discharge [1, 2]. In these regions, approximately half of all deaths among hospitalised children occur after discharge within 6 months post-discharge, often at home or en route to care [2–5]. Risk factors include illness severity at admission, pneumonia, severe anaemia, malnutrition, young age, HIV, prior hospitalisations, discharge against medical advice, and socioeconomic disadvantage [1–5]. The prolonged mortality risk suggests persistent physiological vulnerability beyond the acute illness, underscoring the need to understand modifiable biological mechanisms [6].

Persistent inflammation has been implicated in poor recovery and post-discharge mortality [7–11]. In high-income settings, most sepsis survivors show ongoing inflammation and immune dysregulation, which predict readmission and death [12]. Children admitted with severe malaria exhibit prolonged dysregulation of biomarkers associated with inflammation, coagulation, fibrinolysis, and platelet activation after treatment and recovery [13, 14]. Among sepsis survivors, 15% die within the first year, with an additional 6 − 8% over the next 5 years [15, 16].

In low-resource settings, we previously reported a sepsis-like inflammatory profile among HIV-negative children who died within 2 months after discharge for complicated severe malnutrition (CSM) [17]. Likewise, sustained inflammation, endothelial activation, and enteropathy have been linked to mortality and readmission up to 48 weeks post-discharge in children with CSM in Zambia and Zimbabwe [18]. Higher levels of systemic lipopolysaccharides and inflammatory biomarkers are associated with elevated 90-day mortality among ill hospitalised children in sub-Saharan Africa and South Asia [19].

Early post-discharge deaths may differ biologically from later deaths. In adult intensive care survivors in Brazil, infection-related mortality predominated within 30 days post-discharge, while later deaths were more often linked to ongoing underlying vulnerabilities [20]. Such timing distinctions suggest differing underlying mechanisms, which could inform more targeted post-discharge interventions.

This study investigated whether persistent systemic inflammation and endothelial dysfunction were associated with later post-discharge mortality (later mortality), defined as death occurring 2 − 6 months after hospital discharge, among children previously treated for CSM.

## Methods

### Study design, location and participants

**Fig. 1:**
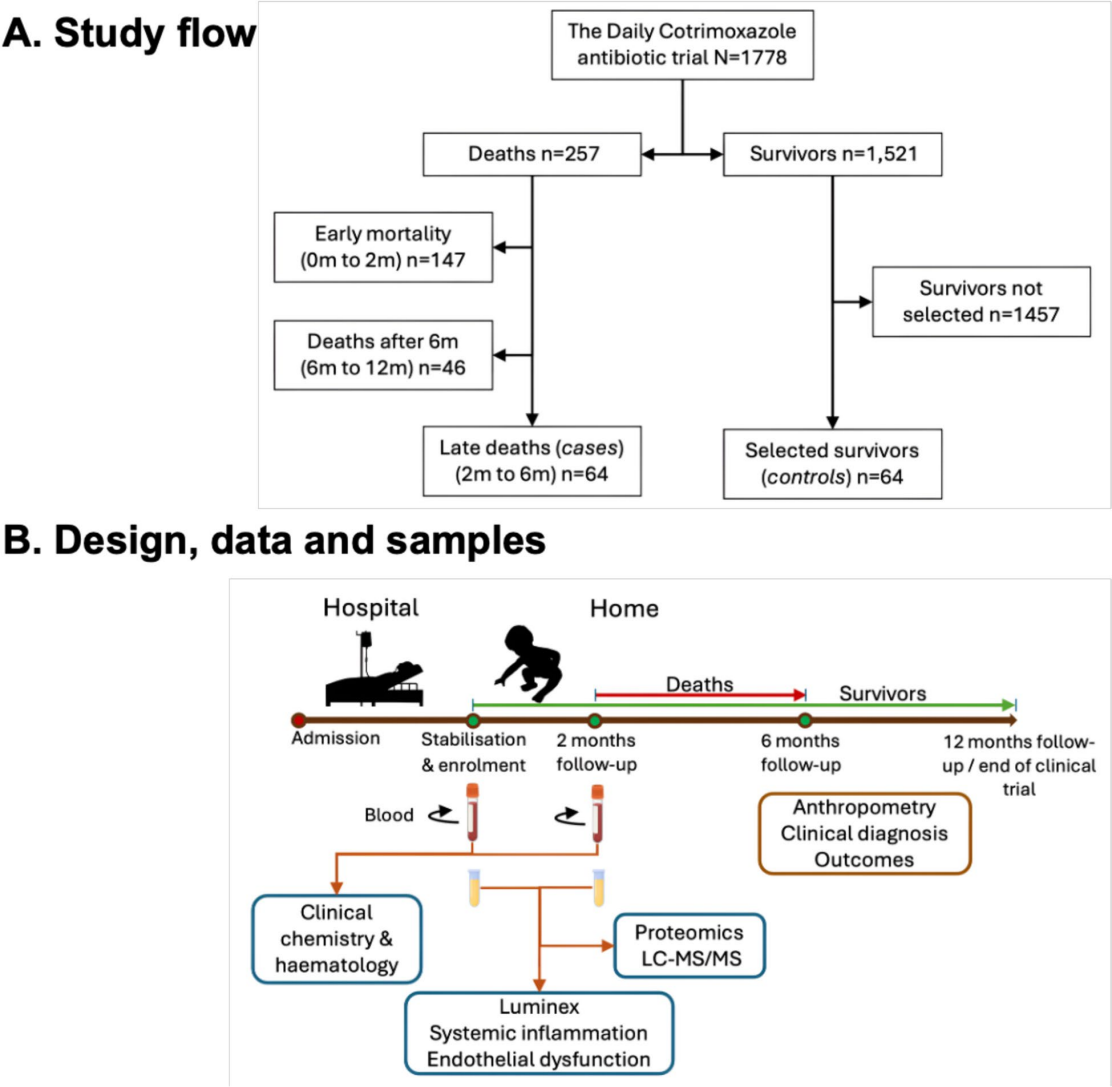
Study design, participants flow, and analytical approach. **A** Flow diagram depicting selection of participants for the late post-discharge mortality (later mortality) analysis. The original trial enrolled 1778 children following stabilisation for complicated severe malnutrition (CSM) and followed for 12 months. Among 257 total deaths, 147 occurred within the first 2 months, 64 between 2 and 6 months (cases in this study), and 46 after 6 months. Sixty-four controls were frequency-matched to cases and had no hospital readmissions during follow-up. **B** Overview of study design and sampling. Blood samples collected at enrolment and 2 months post enrolment were analysed for clinical chemistry, haematology, targeted inflammatory and endothelial biomarkers, and untargeted proteomics via liquid chromatography tandem mass spectrometry (LC–MS/MS). These data were integrated with anthropometric and clinical variables to identify associations with later mortality

This nested case–control study was conducted within a randomised controlled trial evaluating the efficacy of daily co-trimoxazole prophylaxis for 6 months in preventing mortality among HIV-negative children aged 60 days to 59 months treated for CSM (Fig. 1A) [21]. The trial was implemented in four Kenyan hospitals: two urban (Mombasa, Nairobi) and two rural (Kilifi, Malindi). Children were enrolled after completing the ‘stabilisation’ phase of inpatient care, defined by resolution of World Health Organization (WHO)-defined danger signs, improvement in oedema (if present), and ability to tolerate prescribed feeds. Severe malnutrition was classified using WHO criteria: a mid-upper arm circumference (MUAC) < 11.5 cm for children aged 6 − 59 months, < 11 cm for infants aged 2 − 6 months, or the presence of nutritional oedema at any age [22–25]. HIV status was assessed by rapid antibody test and/or HIV-1 PCR. Participants were followed monthly for 6 months, then bimonthly to 12 months.

This analysis primarily used blood samples collected 2 months post-enrolment, with additional analyses using enrolment samples (Fig. 1B). Of the original trial population (*n* = 1778), 147 children (8.3%) died within the first 2 months, 64 (3.6%) died between 2 and 6 months, and 47 (2.6%) died between 6 and 12 months.

### Sample size

We focused on deaths occurring between 2 and 6 months post-discharge (*n* = 64) as later mortality. Sample size for cases was limited by the available cases and available samples at both time-points while budgetary constraints limited the number of controls that could be included. Since this study included all children that died between 2 and 6 months, a formal sample size calculation was not possible. Survivors were frequency matched to cases by site, trial arm, and sex to minimise bias and improve precision. Controls were selected from children who survived the full 12-month follow-up without hospital readmission (*n* = 64), as readmission may have altered mortality risk. Controls were randomly selected at a 1:1 ratio from matched strata.

### Inflammation, coagulation, and proteomics biomarkers

Targeted analysis of 29 cytokines and chemokines (Additional file 1: Table S1) and untargeted plasma proteomics were performed as previously described [17, 26]. Endothelial activation and coagulation markers—thrombomodulin/BDCA-3, angiopoetin-2, D-dimer, and a disintegrin and metalloproteinase with a thrombospondin type-1 motif, member 13 (ADAMTS13)—were measured using a magnetic bead-based assay (R&D Systems, USA) per manufacturer’s protocol.

Biomarker data were log-transformed and autoscaled using mean-centering and standard deviation normalisation (*prep.autoscale* function, R). Outliers (values outside 1.5 × interquartile ranges (IQR)) were capped at the 10th and 90th percentiles. Interleukin 3 was excluded due to high missingness at enrolment, leaving 28 cytokines for analyses (Additional file 2: Tables S2 and S3). For untargeted proteomics, only proteins detected in ≥ 80% of both cases and controls were retained, yielding 374 at 2 months and 423 proteins at enrolment (Additional file 2: Tables S4 and S5). Missing proteomics values were imputated using* k*-nearest neighbours, followed by log transformation, auto-scaling, and batch effect assessment.

### Statistical analysis

Participants’ characteristics at baseline (2 months post-enrolment), including demographic, anthropometric, and full blood count data, were summarised as medians with interquartile ranges (IQR), means with standard deviations (SD) for continuous data, and percentages for categorical variables.

To identify features associated with later mortality, we used conditional Cox regression models via the *coxph* and *Surv* functions in the survival package (v.3.8–3) in R. As frequency matching does not fully control for confounding by design [27], models were adjusted for age, sex, site, randomisation arm and MUAC. Correction for multiple testing was applied using the Benjamini and Hochberg false discovery rate method [28].

## Results

### Participants characteristics

This case–control study included 128 children, with their characteristics at admission, enrolment and at 2 months summarised in Table 1 and Additional file 1: Table S1. Frequency matching ensured a comparable distribution of sex and study site between cases and controls. Compared to controls, cases were younger, more frequently admitted with pneumonia, and had longer hospital stays (*p* < 0.01). At enrolment, cases had lower MUAC, and by 2 months, were more likely to be wasted, underweight, and stunted (all *p* < 0.01).

**Table 1:** Characteristics of study children

Characteristics		Survival status		*p*-value^*2*^
		Cases (*n* = 64)	Controls (*n* = 64)	
**Demographics**				
Age (months)—median (IQR)		7 (5–11)	11 (8–16)	0.002
Sex (male)—no. (%)		31 (48%)	31 (48%)	> 0.9
Hospital stay (days)—median (IQR)		14 (8–25)	10 (7–15)	< 0.001
Site—no. (%)	Kilifi	1 (1.6%)	1 (1.6%)	> 0.9
	Malindi	6 (9.4%)	6 (9.4%)	
	Mbagathi	15 (23%)	15 (23%)	
	Mombasa	42 (66%)	42 (66%)	
**Randomisation arm—no. (%)**				
Cotrimoxazole prophylaxis		31 (48%)	28 (44%)	0.7
**Anthropometry at 2 month**				
Mid upper arm circumference (cm)—median (IQR)		10.7 (9.6 to 11.5)	12.0 (11.3 to 12.7)	< 0.001
Weight-for-age ***Z*** score—median (IQR)		− 4.3 (− 5.6 to − 3.1)	− 3.1 (− 3.7 to − 2.2)	< 0.001
Weight-for-length/height ***Z*** score—median (IQR)		− 3.4 (− 4.4 to − 2.6)	− 2.2 (− 2.9 to − 1.2)	< 0.001
Length/height-for-age ***Z*** score—median (IQR)		− 3.3 (− 4.7 to − 2.2)	− 2.7 (− 3.6 to − 1.5)	0.004
Oedema—no. (%)		1 (1.7%)	1 (1.7%)	> 0.9
**Haematology at 2 months—median (IQR)**				
Haemoglobin g/dL		9.7 (8.3 to 11.4)	10.6 (9.5 to 11.8)	0.026
Platelets count (× 10^3^/μL)		363 (290 to 467)	450 (277 to 566)	0.4
White blood cells count (× 10^3^/μL)		9.8 (6.9 to 12.9)	8.9 (5.7 to 11.2)	0.11
Lymphocytes count (× 10^3^/μL)		4.9 (2.6 to 7.0)	3.6 (2.5 to 6.1)	0.3
Neutrophils count (× 10^3^/μL)		3.5 (2.4 to 4.8)	3.0 (2.1 to 5.1)	0.2

^2^Welch two-sample *t*-test; Pearson’s chi-squared test

Abbreviations: *IQR* interquartile range; α = 0.05

### Changes between enrolment and 2 months

**Fig. 2:**
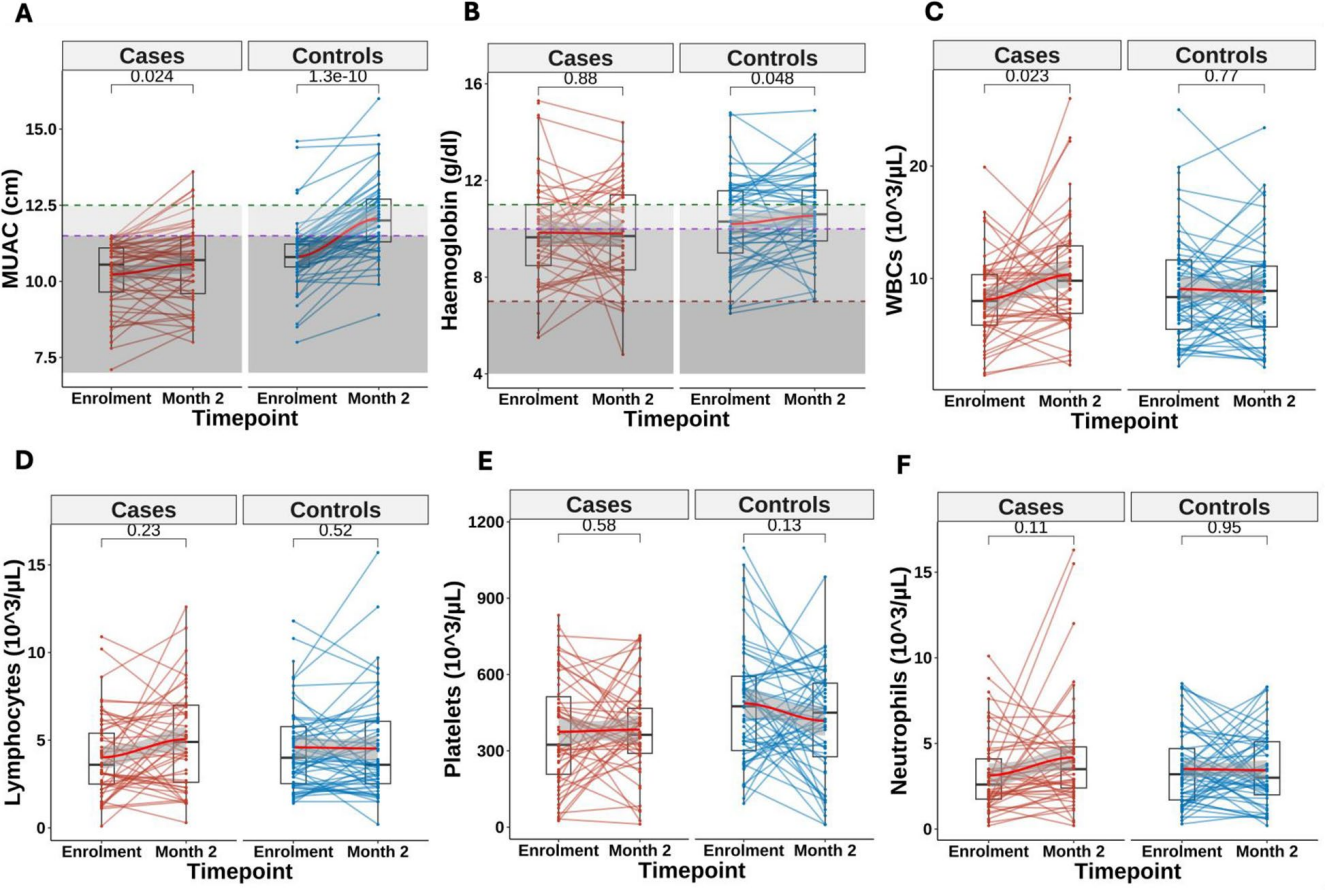
Changes in anthropometric and haematological parameters between enrolment and 2 months post-enrolment among cases and controls. Longitudinal trends in MUAC (**A**), haemoglobin concentration (**B**), white blood cell count (**C**), lymphocyte count (**D**), platelet count (**E**), and neutrophil count (**F**) from enrolment to 2 months post-enrolment. LOESS (Locally estimated scatterplot smoothing) curves (in red) indicate group level trends. In panel **A**, the dashed lines at 12.5 cm (green) and 11.5 cm (purple) denote MUAC thresholds defining no wasting (> 12.5 cm), moderate wasting (11.5 − 12.5 cm), and severe wasting (< 11.5 cm) for children aged 6 − 59 months. In panel **B**, dashed lines at 11 g/dL (green), 10 g/dL (purple), and 7 g/dL (brown) mark haemoglobin thresholds for defining no anaemia (≥ 11 g/dL), mild (10–11 g/dL), moderate (7–10 g/dL), and severe anaemia (< 7 g/dL) in children

From enrolment to 2 months, cases experienced smaller gains in MUAC, weight for age (WAZ) and weight for length/height (WHZ) compared to controls (Fig. 2A, Table 2). Haemoglobin levels remained unchanged in cases but increased among controls (Fig. 2B). Total white blood cell counts increased among cases but remained stable in controls (Fig. 2C, Table 2) primarily driven by lymphocytes and neutrophils (Fig. 2D–F).

**Table 2:** Differences in changes in anthropometric and haematological measurements between enrolment and 2 months post discharge among controls and cases

Change from enrolment to 2 months	Estimate	95% CI	*P* value
Mid upper arm circumference	− 0.22	− 0.30 to − 0.14	< 0.001
Weight-for-age ***Z*** score	− 0.26	− 0.36 to − 0.17	< 0.001
Weight-for-length/height ***Z*** score	− 0.18	− 0.25 to − 0.11	< 0.001
Length/height-for-age ***Z*** score	− 0.05	− 0.15 to 0.05	0.23
Haemoglobin	− 0.02	− 0.06 to 0.03	0.42
Platelets	0.00	− 0.00 to 0.00	0.11
White blood cells	0.02	0.00 to 0.04	0.03
Lymphocytes	0.03	− 0.01 to 0.06	0.12
Neutrophils	0.02	− 0.00 to 0.05	0.11

### Immune dysfunction and pathogen responses are associated with later mortality

**Fig. 3:**
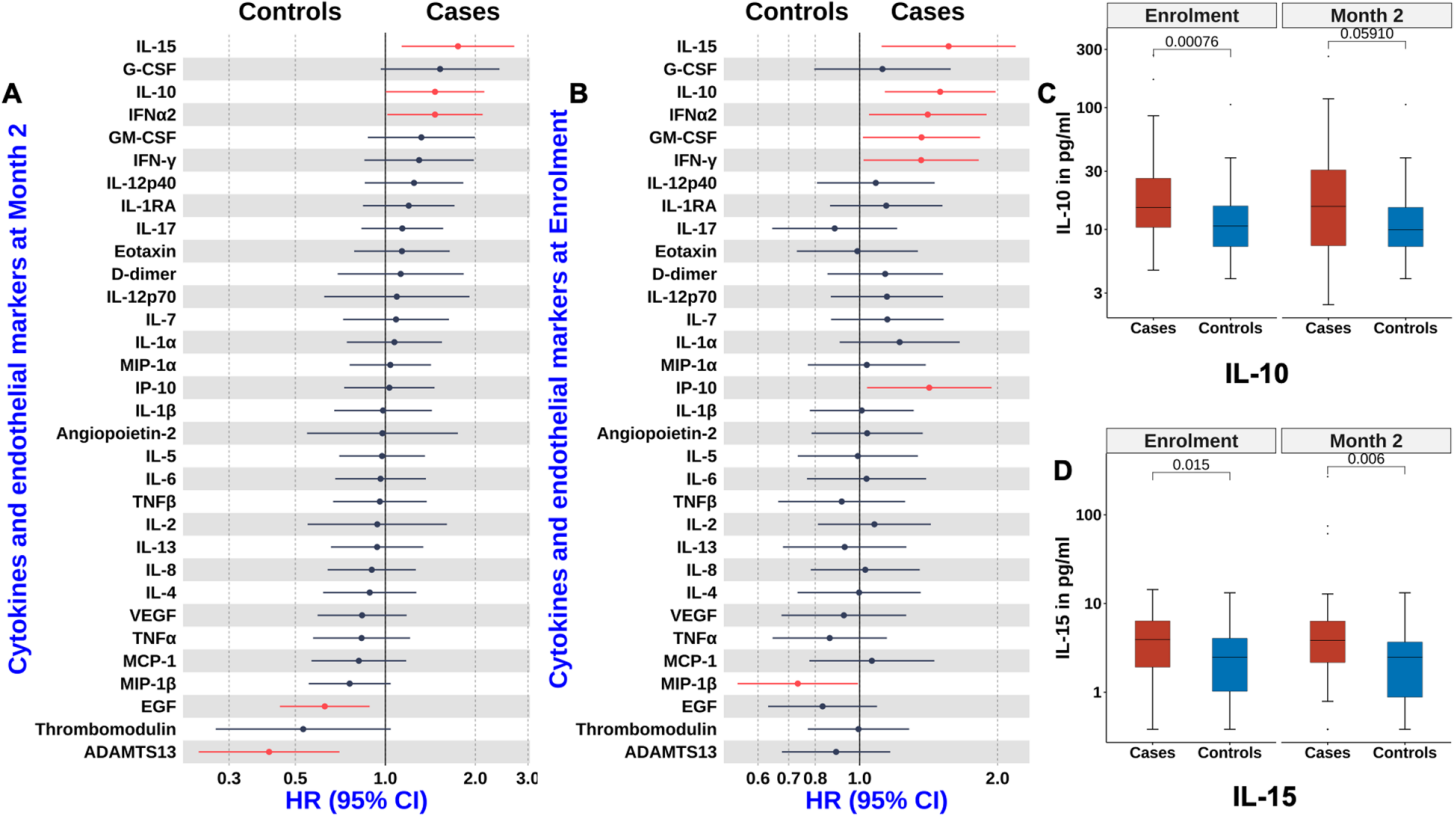
Association between biomarkers of inflammation, coagulation, and later mortality. **A**, **B** Forest plots from adjusted conditional Cox proportional hazards models showing associations between cytokines, chemokines and coagulation markers with later mortality at 2 months post-enrolment (**A**) and at enrolment (**B**). **C**, **D** Boxplots (median and interquartile range) showing the distribution of IL-10 (**C**) and IL-15 (**D**) levels at enrolment and at 2 months post-enrolment. HR = Hazard ratio; CI = Confidence interval

We had previously observed that systemic inflammation was associated with mortality in the first 2 months following discharge [17]. Based on this, we first examined whether biomarkers of systemic inflammation measured 2 months after enrolment were associated with later mortality. Elevated levels of IL-15, IL-10, and interferon alpha 2 (IFNα2) were associated with later mortality (*p* < 0.05, Fig. 3A, Additional file 1: Table S2). Cox proportional hazards models showed that children with high levels of these cytokines had hazard ratios (HR) ≥ 1·5 (Fig. 3A, Additional file 1: Table S2). IL-10 is primarily an anti-inflammatory cytokine that modulates both innate and adaptive immune responses. IL-15 promotes the destruction of infected tissue cells particularly in the context of intracellular pathogens such as viruses. IFNα2 is a type I interferon [29] that also plays a key role in antiviral defense.

We next identified cytokines at enrolment associated with later mortality. Higher levels of IL-15, IL-10, and IFNα2 were associated with later mortality (Fig. 3B, Additional file 1: Table S3). Additionally, elevated IFNγ, IP-10 (CXCL10), and granulocyte–macrophage colony-stimulating factor (GMCSF) were also linked to later mortality. IP-10, a CXC chemokine, promotes chemotaxis, apoptosis, growth inhibition and angiostasis. The consistent association of IL-10 at enrolment and 2 months (Fig. 3C) suggests immune suppression or dysfunction may underlie later mortality. Persistent associations of IL-15 and interferons (Fig. 3B and D) further indicate ongoing infection and immunopathology as contributors to mortality.

**Fig. 4:**
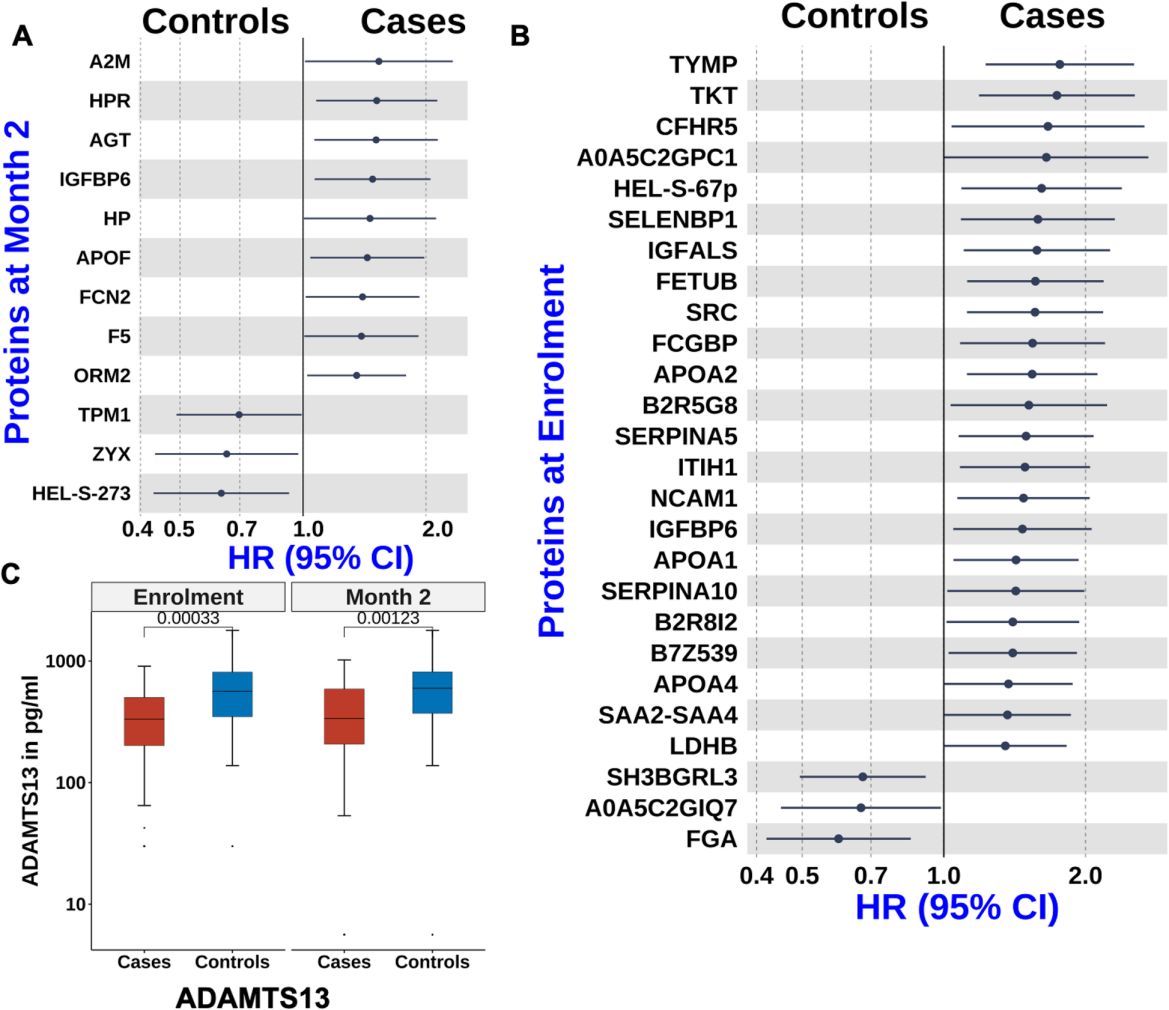
Differential protein expression associated with later mortality. **A**, **B** Forest plots from adjusted conditional Cox proportional hazards models showing proteins identified through untargeted proteomics that were associated with later mortality at 2 months post-enrolment (**A**) and at enrolment (**B**). **C** Boxplots (median and interquartile range) showing the distribution of disintegrin and metalloproteinase with thrombospondin type 1 motif, member 13 (ADAMTS13) levels at enrolment and at 2 months post-enrolment

To complement our targeted analyses and explore additional biological processes associated with later mortality, we performed untargeted proteomic profiling using liquid chromatography-tandem mass spectrometry (LC–MS/MS). At 2 months post-enrolment, cases showed elevated levels of inflammatory proteins orosomucoid-2 (ORM2/α1-acid glycoprotein; AGP), the haemoglobin-binding protein; haptoglobin (HP), and insulin-like growth factor binding protein 6 (IGFBP-6) (Fig. 4A, Additional file 1: Table S4). AGP, HP, and IGFBP-6 are acute phase reactants that increase in response to inflammation. IGFBP-6 also promotes leukocyte chemotaxis, is induced by hypoxia, and helps regulate endothelial inflammation and vascular homeostasis [30, 31].

Proteomics analysis at enrolment revealed that several acute-phase reactants were associated with later mortality, including IGFBP-6, serum amyloid A proteins (B2R5G8, SAA2, SAA4), inter-α-trypsin inhibitors (ITIH1, B7Z539) and complement factor H-related 5 (CFHR5) (Fig. 4B, Additional file 1: Table S5). Fc gamma binding protein (FCGBP), a mucin-like glycoprotein involved in mucosal immunity by inhibiting bacterial attachment and motility, was also elevated in cases. In addition, cases showed increased levels of anti-inflammatory apolipoproteins AI, AII, and AIV (APOAI, APOAII, APOAIV) (Fig. 4A and B). APOAI and APOAII, the main components of high-density lipoproteins, mediate cholesterol efflux and modulate immune cell function [32, 33]. APOAII is primarily synthesised in the liver and intestine and influences responses to lipopolysaccharides and infection [34], while APOAIV, produced in enterocytes, supports lipid metabolism and has antioxidant properties [35, 36].

### Coagulation is associated with later mortality

Given our prior findings that endothelial activation and coagulation were linked to early post-discharge deaths [17, 26], we hypothesised that these processes would also be associated with later mortality. Among the four targeted endothelial and coagulation markers assessed at 2 months, only reduced levels of the disintegrin and metalloproteinase with a thrombospondin type 1 motif, member 13 (ADAMTS13) were associated with increased risk of later mortality (Fig. 3B, Additional file 1: Table S6). ADAMTS13 regulates thrombosis by cleaving von Willebrand factor (vWF) on endothelial surfaces, in circulation, and at sites of vascular injury. In contrast, no targeted coagulation markers at enrolment were associated with later mortality (Fig. 3B, Additional file 1: Table S7).

Untargeted plasma proteomics further identified haptoglobin-related protein (HPR) as associated with later mortality at 2 months (Fig. 4A). HPR binds free plasma haemoglobin released during red blood cell damage, facilitating hepatic iron recycling and protecting against renal toxicity.

At enrolment, several coagulation-related proteins were associated with later mortality, including SERPINA5, SERPINA10, Histidine rich glycoprotein (HRG; B2R8I2), fibrinogen alpha (FGA), and thymidine phosphorylase (TYMP/PD-ECGF) (Fig. 4B, Additional file 1: Table S5). SERPINA5 inhibits protein C and regulates haemostasis and thrombosis, while SERPINA10, a liver-derived protein Z-dependent proteinase inhibitor, inhibits coagulation factors Xa and Xia [37]. HRG interacts with multiple ligands—including heparin, fibrinogen, and immunoglobulin—and participates in coagulation, fibrinolysis, angiogenesis and pathogen clearance [38]. FGA is essential for clot formation and wound healing. TYMP promotes IL-6 production and activation of tissue factor, initiating the extrinsic coagulation cascade [39]. Collectively, these findings indicate that later mortality is associated with sustained dysregulated coagulation.

### Biomarkers of cardiac and vascular physiology are associated with later mortality

To investigate biological processes beyond inflammation and coagulation, we examined proteomic markers related to cardiovascular and cellular function. At 2 months post-enrolment, untargeted plasma proteomics identified angiotensinogen (AGT) as associated with later mortality, consistent with our prior findings [17, 26, 40] (Fig. 4A, Additional file 1: Table S4). AGT is the precursor of angiotensin peptides in the renin–angiotensin–aldosterone system (RAAS), which regulates blood pressure, vascular tone, and cardiac remodelling [41, 42]. Additional proteins associated with later mortality included alpha and beta tropomyosin (TPM1, TPM2), peptidase inhibitor 16 (PI16) and zyxin (ZYX) (Fig. 4A). TPM1 and TPM2 regulate actin-myosin interactions in cardiac muscle contractions. PI16, expressed in cardiomyocytes and regulatory T cells, inhibits chemerin activation, which can induce cardiomyocyte apoptosis [43]. ZYX is a cytoskeletal and adhesion-related protein involved in cellular responses to mechanical stress and is protective against hypertension-induced cardiac dysfunction [44].

Eukaryotic translation initiation factor 5A-2 (EIF5A2) and Rho GDP-dissociation inhibitor 2 (RhoGDI2, ARHGDIB) were also elevated in cases. EIF5A2 regulates translation elongation, mRNA stability, and cell cycle progression, and functions as an oncogene in various cancers [45]. RhoGDI2 is a tumour suppressor involved in cytoskeletal organisation, apoptosis, and T cell function, and is implicated in chronic Chagas cardiomyopathy and suppression of HIV-1 replication [46–48].

At enrolment, cases had higher levels of lactate dehydrogenase (LDH) and transketolase (TKT), metabolic enzymes associated with cellular stress. LDHB is mainly expressed in heart and kidney, converts lactate to pyruvate and is a marker of cell damage and disease severity, while TKT, a key enzyme in the nonoxidative branch of the pentose phosphate pathway, links glucose metabolism with redox balance and biosynthetic processes.

## Discussion

This study investigated whether later post-discharge mortality (later mortality) shares biological features with early deaths in children recovering from CSM. Later mortality cases were younger, often admitted with pneumonia, had longer hospital stays, and were more malnourished with lower haemoglobin at 2 months—consistent with patterns observed in early mortality [17]. Despite apparent clinical recovery, these children exhibited sustained systemic inflammation, immune suppression, and coagulation abnormalities, indicating unresolved subclinical disease.

We previously reported that early post-discharge mortality in this population was marked by a sepsis-like immune profile, including elevated calprotectin, IL-8, IL-15, IP-10, and TNFα at discharge [17]. Here, later mortality was associated with acute phase reactants and proinflammatory mediators, including IL-15, IFNα2, IFNγ, AGP, HP, and SAA – markers commonly elevated in sepsis due to infections or sterile inflammation. These findings align with those of Sturgeon et al., who reported monocyte/macrophage activation markers predicting death or readmission in children with CSM in Zambia and Zimbabwe [18]. In addition, later mortality cases had elevated anti-inflammatory mediators, including IL-10 and apolipoproteins, suggesting concurrent immune suppression. Such dysregulation impairs pathogen clearance and increases susceptibility to secondary infections [19, 49]. Sepsis-induced immunosuppression has been linked to viral reactivation [50], higher rates of positive blood culture [51–53], and expansion of dysfunctional myeloid-derived suppressor cells [54–57]. Malnutrition further compounds immune dysfunction by impairing innate and adaptive responses [58, 59]. Taken together, these findings indicate that later mortality is associated with immune dysfunction characteristic of a sustained sepsis-like response.

Beyond immune dysfunction, coagulation abnormalities were also associated with later mortality. In earlier work, we reported that elevated vWF and decreased SERPIND1 (an anticoagulant) were linked to early mortality [17]. In this study, we identified associations between later mortality and procoagulants FGA and TYMP as well as regulatory proteins ADAMTS13, SERPINA5, SERPINA10, and HRG. Reduced ADAMTS13 is a known driver of thrombotic microangiopathy and sepsis-related hypercoagulability [60]. In sepsis, coagulation and inflammation are closely linked, often resulting in microvascular thrombosis and organ dysfunction [61]. This prothrombotic state is exacerbated by impaired fibrinolysis and suppressed anticoagulant pathways [11, 62–64]. Thromobocytopenia, common in sepsis, may result from decreased platelet production, increased consumption, or sequestration [65–67]. Additionally, depletion of coagulation factors may occur due to impaired hepatic synthesis, vitamin K deficiency, or bleeding – mechanisms also reported in children with undernutrition [68–70].

Our findings highlight biomarkers of cardiovascular homeostasis—AGT, TPM1, TPM2, PI16, and ZYX as being associated with later mortality. AGT, a key component of the RAAS pathway, is implicated in hypertension and endothelial dysfunction [71], and sepsis is a known risk factor for long-term cardiovascular disease in adults [72, 73]. For example, survivors of severe sepsis have a twofold higher risk of cardiovascular events within 1 year post-discharge compared to matched controls [74]. Cardiac atrophy and reduced output are reported in children with severe malnutrition, particularly kwashiorkor [75–77]. However, studies in Kenyan and Malawian children found no significant differences in cardiac function between those with and without CSM, with impairments mainly occurring during acute sepsis [78, 79].

Our findings share features with syndromes described primarily in high-income contexts, including chronic critical illness (CCI), persistent inflammation, immunosuppression, and catabolism syndrome (PICs), post-intensive care syndrome (PICS), proposed to describe persistent illness in high-risk patients. CCI is characterised by prolonged ICU stays, ongoing organ dysfunction, malnutrition, and recurrent infections, often with multidrug-resistant pathogens, leading to gradual deterioration and death [80, 81]. Approximately 30–50% of CCI patients also exhibit features of PICs [82–85]. Paediatric PICs (pPICs) is increasingly recognised [86–89]; in one US study, 47% of children under 21 who died of culture-positive sepsis met ≥ 2 pPICS criteria [87]. Although children in our cohort did not fully meet these syndromic criteria (e.g. we did not observe lymphopenia characteristic of PICs; absolute lymphocyte counts < 1.0 × 10^3^/μL [90]), they displayed overlapping patterns of immune activation, catabolism, and malnutrition. Unlike high-income settings where long-term care is more accessible, children in our study were typically discharged home. Definitions of CCI vary by setting and care context, limiting cross-comparability. PICS, which refers to physical, cognitive, and mental health impairments persisting beyond ICU discharge, has also been linked to systemic inflammation [91, 92], although some studies found no association between early inflammation/coagulation and long-term cognitive outcomes [93]. Further, the Phoenix Sepsis Score to predict poor outcomes is relevant for LMICs, and mortality using this score is higher in low-resource (28.5%) than in high-resource settings (7.1%) [94]. While the score is applicable within hospital settings, it needs validation for identifying children at risk after discharge.

Altogether, our findings indicate later mortality is associated with persistent immune, coagulation, and abnormalities related to cardiac and muscle. Notably, these abnormalities are present in children considered clinically stable at discharge, highlighting the limitations of routine clinical assessments in identifying subclinical risk. Children at highest risk of post-discharge outcomes require more comprehensive diagnostic evaluation and targeted interventions addressing underlying biological pathways. To date, post-discharge strategies in sub-Saharan Africa have largely focused on malaria, anaemia, and acute illness [21, 95–98]. Of these, only post-discharge treatment of malarial anaemia has shown survival benefit and been integrated into WHO guidance. In conditions such as cachexia—characterised by muscle loss unresponsive to nutrition—therapies targeting growth differentiation factor 15 have shown promise in improving appetite, weight gain, and activity [99] though high cost limits feasibility in low-resource settings. Immune modulating micronutrients (e.g. vitamins A, C, D, iron, selenium, zinc) warrant evaluation for their potential to enhance immune recovery. Additionally, closer monitoring of weight gain and anaemia, especially among younger children with longer hospitalisation, may help reduce adverse outcomes. However, post-discharge mortality reflects multifactorial risks. Broader strategies are needed, including caregiver education, household-level interventions, improved health-seeking behaviours and systems-level support [2]. In resource-constrained settings, targeting the highest-risk children may offer the most efficient path to improving long-term survival.

This was a secondary analysis of data and samples from a prior clinical trial with minimal loss to follow-up. However, the targeted panel was limited, and more advanced proteomic and metabolomic technologies now available could provide deeper insights. While we focused on systemic host responses, other factors—such as micronutrient deficiencies, co-infections, enteropathy and gut microbiota alterations—may also contribute to mortality, as we have recently shown [19] but were not assessed. The sample size, constrained by the original study design, limited statistical power to detect smaller effect sizes and to robustly assess longitudinal interactions. Additionally, limited collection of clinical and socioeconomic data limited our ability to evaluate their contribution to post-discharge outcomes.

## Conclusions

This study suggests that later mortality among children recovering from CSM is associated with persistent systemic inflammation, immune suppression, and coagulation abnormalities—despite apparent clinical recovery. While these subclinical disturbances, undetectable through routine clinical assessments, require external validation, they highlight the need for improved risk stratification tools and targeted interventions. Existing post-discharge strategies in sub-Saharan Africa remain limited in scope, and few have shown survival benefit. Future approaches may consider integrating biomarker-driven diagnostics, immune and nutritional support, and community-based follow-up to address the multifactorial nature of post-discharge mortality. Targetting high-risk children through tailored, resource-sensitive interventions may offer an effective path to reduce long-term mortality and morbidity in this vulnerable population.

## Supplementary Information

Additional file 1: Table S1 – The enrolment characteristics of study children stratified by survival status.

Additional file 2: Tables S2 to S7 – Results for the differential expression and Cox regression analysis at 2 months post-enrolment and at enrolment. Table S2-Results for the 29plex Luminex panel at 2 months post-enrolment. Table S3-Results for the 29plex Luminex panel at enrolment. Table S4-Results for the untargeted proteomics at 2 months post-enrolment. Table S5-Results for the untargeted proteomics at enrolment. Table S6-Results for the endothelial biomarkers at 2 months post-enrolment. Table S7- Results for the endothelial biomarkers at enrolment.

## Data Availability

The data and analysis code that support the findings of this study are archived on the Harvard Dataverse (10.7910/DVN/FB5XQR) [100]. Access to these data requires submission of a formal request for consideration by our Data Governance Committee. Email completed data request form to the Data Governance Committee at dgc@kemri-wellcome.org. The mass spectrometry raw files generated and analysed in the current study have been deposited to the ProteomeXchange Consortium (PXD067346), via the MassIVE partner repository (MSV000098824), under the following title: Persistent immune, coagulation and cardiac dysregulation predict late post discharge mortality in children with severe malnutrition.

## Abbreviations

ADAMTS13: A disintegrin and metalloproteinase with thrombospondin type 1 motif, 13
AGT: Angiotensinogen
CSM: Complicated severe malnutrition
CCI: Chronic critical illness
CFHR5: Complement factor H-related 5
EIF5A2: Eukaryotic translation initiation factor 5A-2
FGA: Fibrinogen alpha
GMCSF: Granulocyte-macrophage colony-stimulating factor
HPR: Haptoglobin-related protein
IFN: Interferon
IL: Interleukin
IQR: Interquartile ranges
LDH: Lactate dehydrogenase
Later mortality: Late post-discharge mortality
MUAC: Mid-upper arm circumference
RhoGDI2: Rho GDP-dissociation inhibitor 2
SERPINA: Serpin Family A Member
SD: Standard deviation
TPM: Tropomyosin
TKT: Transketolase
TYMP: Thymidine phosphorylase
vWF: Von Willebrand factor
WAZ: Weight for age
WHO: Word Health Organisation
WHZ: Weight for length/height
