# Supplementary material for: Persistent immune, coagulation and cardiac dysregulation are correlated with later post-discharge mortality in children with severe malnutrition
Source: BMC Med. 2026 Jan 22;24:100. doi: 10.1186/s12916-026-04647-9 (PMC12911261; doi:10.1186/s12916-026-04647-9)
Supplement: Supplementary file 1 — Additional file 1: Table S1 – The enrolment characteristics of study children stratified by survival status. [file 12916_2026_4647_MOESM1_ESM.docx]

**Persistent immune, coagulation and cardiac dysregulation are correlated with later post-discharge mortality in children with severe malnutrition**

**Authors**

Brenda Kamau^1,#^, Evans O. Mudibo^1,2,3,#^, Cecillia Wechessa^1^, Elisha Omer^1,2^, Bonface M Gichuki^1,2,4^, David M. Mburu^5^, Laura Mwalekwa^1,2,6^, Molline Timbwa^1,2,7^, Johnstone Thitiri^1,2^, Moses M. Ngari^1,2^, James A. Berkley^1,2,8,*^, James M. Njunge^1,2,9,*^.

**Affiliations**

1. KEMRI-Wellcome Trust Research Programme; Kilifi, Kenya.
2. The Childhood Acute Illness and Nutrition Network; Nairobi, Kenya.
3. Division of Human Nutrition and Health, Wageningen University and Research; Wageningen, Netherlands.
4. Host-Microbiota Interactions Lab, Wellcome Sanger Institute, Hinxton, UK
5. Pwani University
6. Coast General Hospital, Mombasa, Kenya.
7. Mbagathi County Hospital, Nairobi, Kenya.
8. Center for Tropical Medicine and Global Health, Nuffield Department of Medicine, University of Oxford; Oxford, UK.
9. Gonville and Caius College, University of Cambridge, Cambridge, UK.

^*^ Corresponding author ([jnjunge@kemri-wellcome.org](mailto:jnjunge@kemri-wellcome.org))

^#^ Equal contributions.

| Table S1. Characteristics of study children | | | | |
| --- | --- | --- | --- | --- |
| Characteristics | | Survival status | | p-value*^2^* |
|  |  | Cases (n=64) | Controls (n=64) | *^-^* |
| Demographics at enrolment | | | | |
| Age (months) – median (IQR) | | 7 (5 - 11) | 11 (8 - 16) | 0.002 |
| Sex (male) – no. (%) | | 31 (48%) | 31 (48%) | >0.9 |
| Site – no. (%) | Kilifi | 1 (1.6%) | 1 (1.6%) | >0.9 |
|  | Malindi | 6 (9.4%) | 6 (9.4%) |  |
|  | Mbagathi | 15 (23%) | 15 (23%) |  |
|  | Mombasa | 42 (66%) | 42 (66%) |  |
| Randomisation arm | | | | |
| Cotrimoxazole prophylaxis – no. (%) | | 31 (48%) | 28 (44%) | 0.7 |
| Clinical illness at admission – no. (%) | | | | |
| Pneumonia | | 50 (78%) | 33 (52%) | 0.003 |
| Diarrhoea | | 24 (38%) | 35 (55%) | 0.076 |
| Shock | | 2 (3.1%) | 6 (9.4%) | 0.3 |
| Cerebral palsy | | 7 (11%) | 2 (3.1%) | 0.2 |
| Anthropometry at enrolment | | | | |
| Mid upper arm circumference – median (IQR) | | 10.6 (9.6 to 11.1) | 10.8 (10.5 to 11.2) | 0.003 |
| Weight-for-age Z score – median (IQR) | | -4.2 (-5.1 to -3.1) | -3.9 (-4.5 to -3.2) | 0.054 |
| Weight-for-length/height Z score – median (IQR) | | -3.6 (-4.3 to -2.7) | -3.5 (-4.2 to -2.7) | >0.9 |
| Length/height-for-age Z score – median (IQR) | | -2.9 (-4.3 to -1.7) | -2.6 (-3.6 to -1.3) | 0.043 |
| Oedema – no. (%) | | 4 (6.3%) | 10 (16%) | 0.2 |
| Haematology at enrolment – median (IQR) | | | | |
| Haemoglobin (g/dL) | | 9.7 (8.5 to 11.0) | 10.3 (8.8 to 11.6) | 0.4 |
| Platelets count (x10^3^/μL) | | 324 (208 to 513) | 475 (301 to 593) | 0.008 |
| White blood cells count (x10^3^/μL) | | 8.0 (5.9 to 10.4) | 8.5 (5.7 to 11.8) | 0.12 |
| Lymphocytes count (x10^3^/μL) | | 3.6 (2.5 to 5.4) | 4.0 (2.5 to 5.8) | 0.2 |
| Neutrophils count (x10^3^/μL) | | 2.6 (1.8 to 4.1) | 3.2 (1.7 to 4.8) | 0.2 |
| ^2^Welch Two Sample t-test; Pearson's Chi-squared test. Abbreviations: IQR = Interquartile range; α = 0.05 | | | | |
